# Pre‐analytical pitfalls: How blood collection tubes influence exercise‐induced cell‐free DNA concentrations

**DOI:** 10.1113/EP092284

**Published:** 2025-03-03

**Authors:** Kira Enders, Barlo Hillen, Nils Haller, Alexandra Brahmer, Vincent Weber, Perikles Simon, Elmo W. I. Neuberger

**Affiliations:** ^1^ Department of Sports Medicine, Disease Prevention and Rehabilitation Johannes Gutenberg University Mainz Mainz Germany; ^2^ Department of Sport and Exercise Science University of Salzburg Salzburg Austria

**Keywords:** blood biomarker, blood collection tubes, cell‐free DNA, circulating DNA, exercise, pre‐analytical factors, running

## Abstract

Circulating cell‐free DNA (cfDNA) is a promising biomarker for physiological stress, including exercise‐induced responses. However, the lack of standardization in blood collection tubes (BCTs) for quantification of cfDNA hampers inter‐study comparisons. In this study, we assessed the impact of different BCTs on exercise‐induced cfDNA dynamics. Eleven participants [25 (SD 2.3) years of age] performed three different treadmill exercise protocols, including an all‐out test and combinations of constant and interval load. Blood samples were collected before, 5 min and 30 min post‐exercise using EDTA, lithium–heparin (LH) and serum BCTs. Concentrations of cfDNA were quantified using quantitative PCR. The cfDNA increased significantly across all protocols and BCTs. A significant effect of BCT on cfDNA concentrations (*P* = 0.034) was found, with serum showing higher concentrations than EDTA and LH. Although absolute differences from pre‐ to post‐exercise were comparable across BCTs (*P* = 0.476), fold changes differed significantly (*P* = 0.012), with the highest observed in EDTA and the lowest in serum. Bland–Altman analyses demonstrated better agreement between EDTA and LH compared with serum. Significant correlations of cfDNA with energy expenditure and peak oxygen uptake were found. These correlations were stronger in EDTA and LH than in serum. Our findings highlight the crucial influence of BCT choice on cfDNA measurements in exercise settings. Given that EDTA and LH reflected exercise load better, they could be preferred for exercise physiology research. This work underscores the need to account for the choice of BCT to improve data comparability across studies. Additionally, these findings might have broader implications for clinical settings where cfDNA is used as a biomarker.

## Abstract (highlights)

- **What is the central question of this study?**
  This work examines how different blood collection tubes influence cell‐free DNA concentrations in standardized exercise settings. Additionally, correlations of exercise‐induced cell‐free DNA with exercise‐related parameters were investigated.
- **What is the main finding and its importance?**
  All blood collection tubes used (EDTA, lithium–heparin and serum) show typical exercise‐induced increases in cell‐free DNA. Fold changes and absolute concentrations differ between blood collection tubes, but absolute pre to post differences are comparable. EDTA and lithium–heparin show better agreement than serum with either EDTA or lithium–heparin. In addition, stronger correlations with exercise‐related parameters were observed.

## 1 INTRODUCTION

Exercise physiologists continue to seek suitable biomarkers for objective monitoring of exercise load, optimizing load management in athletes and enhancing their performance. Amongst potential candidates, circulating cell‐free DNA (cfDNA) has shown a promising potential to reflect physiological responses to exercise for several reasons (Breitbach et al., 2012; Fridlich et al., 2023). Increases in cfDNA have been observed consistently across diverse exercise modalities, including acute aerobic exercise (Beiter et al., 2011), resistance exercise (Atamaniuk et al., 2010) and regular soccer season activities (Gentles et al., 2015). Additionally, previous research has demonstrated that increases in cfDNA are closely associated with exercise intensity, duration and modality (Breitbach et al., 2012; Haller et al., 2017; Nogiec et al., 2024), with studies reporting increases of up to 20‐fold in ultra‐marathon runners (Atamaniuk et al., 2008). In comparison to traditional markers of muscle damage, such as creatine kinase, uric acid or C‐reactive protein, which exhibit delayed kinetics, cfDNA responds rapidly to exercise (Breitbach et al., 2012). Its concentrations have been shown to increase immediately owing to neutrophil activation and aseptic inflammation triggered by physical activity (Fatouros et al., 2006; Fridlich et al., 2023; Neuberger et al., 2022). The potential of cfDNA measurements to serve as a biomarker for exercise load is also linked to their accuracy in reflecting exercise‐related parameters (Haller et al., 2023). Previous ‘all‐out’ exercise studies have observed correlations between cfDNA concentrations and heart rate, lactate, oxygen uptake (${\dot{V}}_{O_{2}}$), energy expenditure, exercise duration and the rating of perceived exertion (RPE) (Breitbach et al., 2014). In endurance running, Nogiec et al. (2024) observed a significant correlation between cfDNA and energy expenditure. These rapid, sensitive and load‐dependent responses to physical strain position cfDNA as a valuable tool for objective monitoring of internal load, offering the potential to enhance athlete workload management and optimize performance (Haller et al., 2023).

Despite the great potential of cfDNA, its application has so far been limited to research settings. The lack of consistent guidelines for measuring cfDNA in exercise contexts has resulted in varying methodologies being adopted by different research groups. However, to ensure reliable comparisons between studies, accurate and standardized measuring techniques need to be adopted. Not only measurement‐related factors, such as the method of quantification (Peng et al., 2024), but also pre‐analytical variability (Ammerlaan & Betsou, 2019) might affect the outcome. In this respect, the process of blood collection is known to have a major impact on cfDNA concentrations (Trigg et al., 2018; Ungerer et al., 2020). As yet, there is no consensus about the usage of specific blood collection tubes (BCTs) for cfDNA analysis in the field of exercise physiology research. Most working groups use EDTA plasma for cfDNA quantification (e.g., Atamaniuk et al., 2008; Beiter et al., 2014; Ferrandi et al., 2018; Fridlich et al., 2023; Haller et al., 2018; Hummel et al., 2018; Neuberger et al., 2022; Stawski et al., 2017; Ungerer et al., 2020; Walczak et al., 2021), whereas several other working groups have opted for serum as the medium for cfDNA analysis (e.g., Andreatta et al., 2018; Belcher et al., 2019; Shishikura et al., 2021; Velders et al., 2014). In the 1990s, lithium–heparin (LH) tubes were thought to inhibit PCR (Beutler et al., 1990; Jung et al., 1997; Yokota et al., 1999). However, more recent studies in non‐exercise contexts have demonstrated that LH tubes, in principle, can be used for quantification of cfDNA (Lam et al., 2004; van Ginkel et al., 2017). Thus far, comparing cfDNA concentrations in exercise studies using different BCTs remains challenging, because a systematic comparison between different BCTs and their influence on cfDNA concentrations and on exercise‐induced increases in concentration has not yet been conducted.

In non‐exercise settings, previous studies have already shown that serum yields higher cfDNA concentrations than EDTA or LH, with differences ranging from 5‐ to 20‐fold (Ammerlaan & Betsou, 2019; Lee et al., 2001; Martignano, 2019; Trigg et al., 2018). Higher cfDNA concentrations in serum samples probably result from contamination by genomic DNA, because the clotting process lyses white blood cells, thus releasing nuclear fragments (Lee et al., 2001). In contrast, no conclusive evidence was found when comparing cfDNA concentrations between EDTA and LH samples. Lam et al. (2004) showed no significant differences between LH and EDTA when blood processing was done within 6 h after venesection. In another study, van Ginkel et al. (2017) reported significantly lower concentrations in LH than in EDTA. Considerably, these studies have typically focused on a single time point for blood sampling. As a result, these studies do not provide insights into the comparability of the kinetics of cfDNA increases in different BCTs. It is therefore still unknown to what extent the type of BCT might have an effect on exercise‐induced increases in cfDNA. In addition to the impact of the BCT on cfDNA concentrations, it is crucial to examine the relationship between exercise‐related physiological parameters and cfDNA concentration measured in different BCTs. The previously presented correlations were based solely on EDTA plasma samples. Hence, it remains unclear whether LH or serum is equally suitable for detecting them.

In this study, we investigated the impact of EDTA, LH and serum BCTs on the cfDNA concentration measured with direct quantitative PCR (qPCR) before and after different acute exercise settings and examined associations between different exercise‐related parameters and the cfDNA measured in the respective BCTs. The results of our study will allow a deeper understanding of cfDNA concentrations measured with different BCTs, with the aim of improving comparability of cfDNA analysis in different studies.

## 2 MATERIALS AND METHODS

### 2.1 Ethics

Experimental procedures were approved by the human ethics committee of Rhineland‐Palatinate (ethical approval identifier: ‘2021‐15713’) and conformed to the *Declaration of Helsinki* of the World Medical Association. Additionally, the study has been registered in a clinical trial registry (clinical trial number: DRKS00029114). Participants were informed orally and in writing about the procedures and the aim of the study and gave written consent to participate.

### 2.2 Study design

**FIGURE 1:**
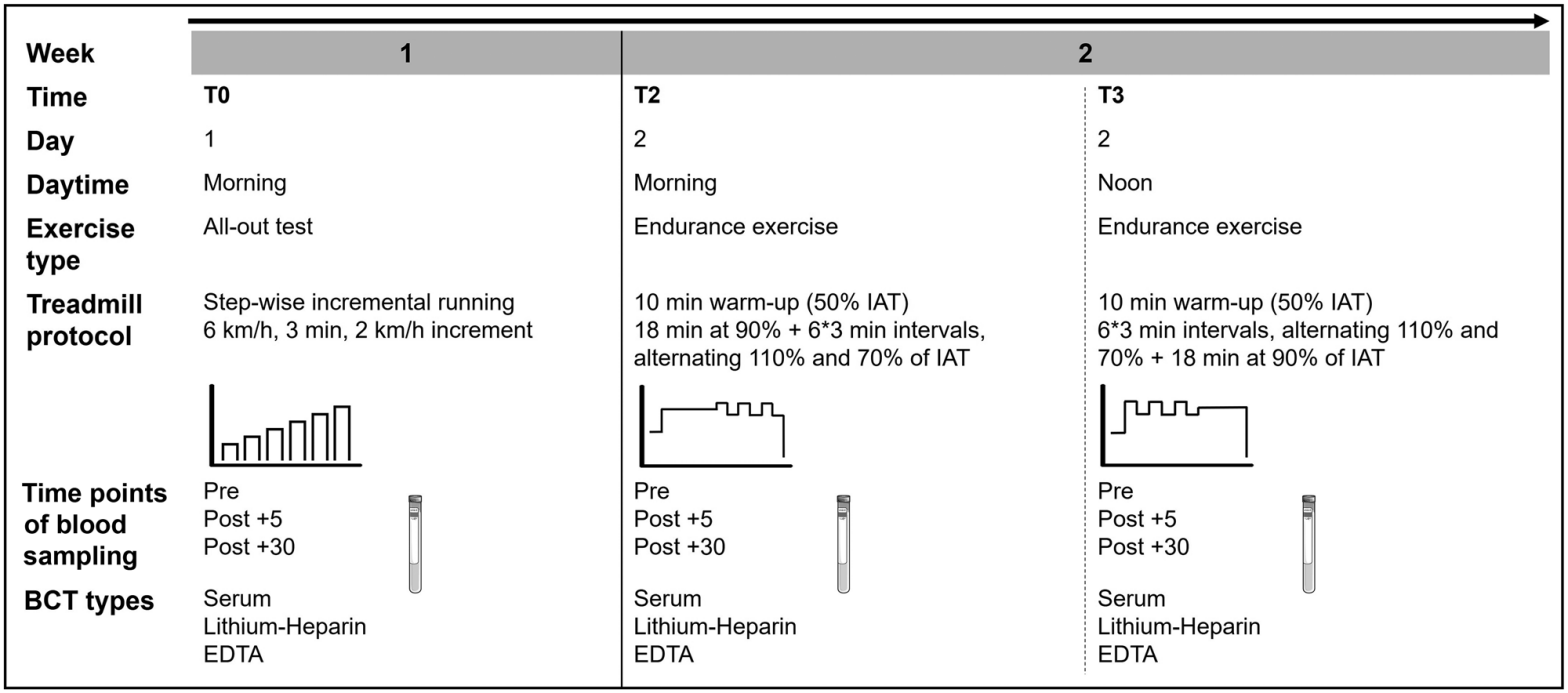
Study design. Every participant made two visits, one in week 1 and one in week 2. They underwent different exercise protocols. At T0, T2 and T3 blood was sampled at three time points: before the onset of exercise (pre); within 5 min after cessation of the exercise (post +5); and 30 min after cessation of the exercise (post +30). At every time point, blood was sampled into serum, LH and EDTA BCTs. Abbreviations: BCT, blood collection tube; IAT, individual anaerobic threshold; LH, lithium–heparin.

Fourteen (six male and eight female) participants were recruited for this study. Inclusion criteria required participants to have a physical activity rating (PA‐R10 scale) of >6 out of 10 (defined by regular physical activity of moderate to high intensity, ∼30–60 min on ≥3–5 days a week) (George et al., 1997), without acute injuries or health restrictions. All participants completed three different treadmill exercise protocols during two visits, during which venous blood was collected, processed and analysed as detailed below. The participants were asked not to exercise for 24 h prior to the visits. Figure 1 outlines the study design.

At T0, participants underwent a stepwise incremental running test until voluntary exhaustion. Participants started at 6 km/h, with an increment of 2 km/h every 3 min. After completing every stage, capillary blood was sampled from the earlobe to measure lactate concentrations. The individual anaerobic threshold (IAT) was determined for each individual using the minimal lactate equivalent +1.5 mmol/L (Roecker et al., 1998). Venous blood was collected pre‐ and post‐exercise (+5 and +30 min) using three different BCTs as described in the section 2.3.

On the second visit (1 week after T0), participants performed two treadmill runs combining constant and interval loads. These sessions, referred to as T2 and T3, both started with a standardized 10 min warm‐up at 50% of their IAT. At T2, participants first performed an 18 min continuous run at 90% of their IAT, followed by six 3 min interval bouts alternating between 110% and 70% of their IAT. At T3, the order of the exercise components was reversed. Participants started with six 3 min interval bouts at 110% and 70% of their IAT, followed by the 18 min continuous run at 90% of their IAT (Figure 1). Both sessions (T2 and T3) lasted a total of 46 min. Similar to T0, venous blood samples were collected pre‐ and post‐exercise (+5 and +30 min) using all BCTs. Between T2 and T3, there was a resting period of ∼3 h to warrant a return to baseline of the cfDNA concentration in the pre‐exercise values of T3 (Breitbach et al., 2014; Yamamoto et al., 2025).

Air volume and gases were measured continuously using a breath‐by‐breath method (Ergostik, Geratherm Respiratory, Bad Kissingen, Germany), allowing us to calculate the energy expenditure, oxygen uptake (${\dot{V}}_{O_{2}}$) and peak oxygen uptake (${\dot{V}}_{O_{2}\mathrm{peak}}$) with spiroergometry software (BlueCherry, Geratherm Respiratory). Heart rate was measured continuously using a 12‐channel ECG. Body core temperature was measured via telemetric pills (Telemetric System for Continuous Gastrointestinal Temperature Monitoring, BodyCAP, Hérouville Saint‐Clair, France). The RPE was assessed every 3 min using a scale of 6–20 (Borg, 1998). Mean heart rate, mean ${\dot{V}}_{O_{2}}$, mean body temperature and mean RPE were calculated for the duration of each exercise protocol.

### 2.3 Blood collection and processing

K_3_‐EDTA (2.6 mL), LH (2.7 mL, 16 IU/mL) and serum (2.7 mL, Z‐Gel clot activator) Monovettes (Sarstedt AG & Co. KG, Nümbrecht, Germany) were used in this study. As recommended by the World Health Organization (2010) and the manufacturer's guidelines, the following order of BCTs was followed for all blood sampling: serum, LH and EDTA. Lactate concentrations were measured with the Biosen 5130 (EKF Diagnostics, Magdeburg, Germany), and analyses were conducted using the software Winlactat v.4.0 (Mesics, Münster, Germany).

After blood collection, the samples were inverted thoroughly to distribute the anticoagulants and promptly taken to the laboratory. EDTA and LH tubes were processed within 5 min after the collection. Serum tubes were stored for 30 min to allow blood coagulation prior to processing. Centrifugation was performed at 2500*g* at +22°C for 15 min. Plasma (1.1 mL) was taken and centrifuged a second time for 15 min at 2500*g*. All aliquoted plasma and serum samples were stored at −80°C.

### 2.4 Cell‐free DNA measurement

The cfDNA measurement used in this study corresponds to the assay described and validated by Neuberger et al. (2021). In short, plasma and serum were thawed at room temperature (22°C) and diluted 1:10 using UltraPure DNase/RNase‐Free H_2_O (Invitrogen, Waltham, MA, USA). Two microlitres of the dilution was mixed with 1 µL of primer mix and 12 µL of master mix to prepare triplicates with a final volume of 5 µL. The final concentration of the primers was 140 nM for both primers, forward and reverse. The primer sequences were 5′‐TGCCGCAATAAACATACGTG‐3′ and 5′‐GACCCAGCCATCCCATTAC‐3′, priming a 90 bp fragment of DNA from the L1PA2 region. The final concentrations of the master mix components in the PCR were 1.2 × Hifi Buffer (Bioline), 0.3 mM dNTPs (Carl Roth), 0.15 × SYBR Green nucleic acid gel stain (from a ×10,000 stock solution, Sigma‐Aldrich) and 0.04 u/µL of Velocity Polymerase (Bioline). After preparing the triplets in the well plate, the plate was centrifuged at 1600 *g* for 2 min.

The qPCR was carried out with a Bio‐Rad CFX384 system (Bio‐Rad, Hercules, CA, USA) in adherence to the following protocol. The first step was 98°C for 2 min, followed by 35 cycles with denaturation at 95°C for 10 s and annealing at 64°C for 10 s. The plate read was included in the annealing phase of each cycle. For the melt curve analysis, the starting temperature was 70°C, incrementing by 0.5°C every 10 s up to 95°C.

### 2.5 Statistical analyses

The collected data of the Bio‐Rad CFX384 system were analysed with the software Bio‐Rad CFX Manager v.3.1 (2012, file version: 3.1.1517.0823). The raw data of the cq‐values were calibrated using samples with known cq‐values and thus known cfDNA concentrations. The calculation used to determine the cfDNA concentrations (in nanograms per millilitre) on the basis of the cq‐values is described further by Neuberger et al. (2021). All calculations were performed within an Excel file (Microsoft Office, 2021). Statistical analyses and figure illustrations were conducted using RStudio (RStudio Team, 2024, v.2024.04.1, Boston, MA, USA) with the following packages: *rstatix* (v.0.7.2), *ggplot2* (v.3.5.1), *BlandAltmanLeh* (v.0.3.1), *corrplot* (v.0.85) and *tidyverse* (v.2.0.0).

Fold changes were calculated by dividing the post‐exercise (+5 and +30) values by the pre‐exercise values (hereafter referred to as fold change +5 and fold change +30). Absolute differences in cfDNA levels were calculated by subtracting the pre‐exercise values from the post‐exercise values (+5 and +30; hereafter referred to as absolute difference +5 and absolute difference +30). The cfDNA concentrations were log‐normalized, ensuring a normal distribution for time points and BCTs within the three exercise protocols. To compare cfDNA concentrations, fold changes and absolute differences measured with the different BCTs for the different exercise protocols, repeated‐measures ANOVAs were performed with the interaction of time (i.e., T0, T2 and T3), time point (i.e., pre, post +5 and post +30) and BCT set as within‐subject factors (three‐way ANOVA). The assumption of sphericity was assessed using Mauchly's test. When sphericity was violated, the Greenhouse–Geisser correction was applied. *Post hoc* analyses were performed using pairwise comparisons with Bonferroni correction. Correlation between the absolute differences in the BCTs was calculated using Spearman correlation. Bland–Altman plots were created to compare the agreement of the absolute differences. Limits of agreement were calculated using 1.96 × SD as recommended (Bland & Altman, 1986). For the correlation of cfDNA differences and fold changes with exercise parameters, Spearman correlation was conducted. The α‐level of significance was set to *P* < 0.05 for all statistical analyses.

## 3 RESULTS

### 3.1 Characteristics of study participants

Out of 14 participants, two participants discontinued the study after T0 owing to medical concerns (not fully recovered from recent COVID‐19 infection). In addition, one participant was excluded because blood sampling at T3 was not possible. Thus, 11 participants (five female, six male) completed all exercise tests and were included in the analyses. Anthropometric data of the study participants are provided in Table 1.

**TABLE 1:** Anthropometric data of the study participants.

Variable	*n*	Mean	SD
Age, years	11	25	2.3
Height, cm	11	175	5
Body mass, kg	11	68.4	8.3
PA‐R10, scale 1–10	11	8.4	1.4
${\dot{V}}_{O_{2}\mathrm{peak}}$, ml/min/kg	11	52.9	7.7

Abbreviations: PA‐R10, physical activity rating; ${\dot{V}}_{O_{2}\mathrm{peak}}$, peak oxygen uptake.

### 3.2 Cell‐free DNA concentrations in different BCTs

The cfDNA concentrations for all exercises, time points and BCTs are shown in Table 2. In all three BCTs, the lowest cfDNA concentrations were measured before the onset of exercise. In EDTA, the mean pre‐exercise concentration ranged from 13 to 14.8 ng/ml. In comparison, the mean pre‐exercise concentration of LH samples ranged from 18.2 to 26.2 ng/ml. In serum, the highest mean pre‐exercise concentrations were observed, with a range of 41.9 to 77.1 ng/ml. Significant increases were measured after exercise in all BCTs, with cfDNA concentrations increasing up to 186.3 ng/ml in EDTA, 198 ng/ml in LH and 222.3 ng/ml in serum samples. Thirty minutes after exercise, the concentrations were still elevated in comparison to the pre‐exercise values in most observations.

**TABLE 2:** Cell‐free DNA concentrations of the participants.

Time	Time point	*n*	EDTA (ng/ml)	LH (ng/ml)	Serum (ng/ml)
T0	Pre	11	13.8 (6.2)	26.2 (25.1)	56.3 (37)
	Post +5	11	131.8 (63.2)^****^	124.8 (56.3)^***^	176.6 (64.7)^***^
	Post +30	11	33.7 (18.1)^****^	38.3 (18.2)	89.1 (41.2)^*^
	Absolute difference +5	11	118 (59.8)	98.6 (69.7)	120.3 (72.2)
	Fold change +5	11	9.8 (4.2)	7.1 (4.6)	4.5 (3.4)
	Absolute difference +30	11	19.8 (13.8)	12.1 (34.6)	32.7 (39.9)
	Fold change +30	11	2.4 (0.9)	2.1 (1.4)	2.1 (1.6)
T2	Pre	11	14.8 (6)	18.2 (8.1)	41.9 (23.2)
	Post +5	11	129.9 (84.3)^****^	134.4 (86.3)^****^	160.2 (86.7)^***^
	Post +30	11	54 (43.9)^***^	57.9 (46)^***^	73.1 (43.5)^*^
	Absolute difference +5	11	115.1 (81.9)	116.1 (84.1)	118.3 (92.5)
	Fold change +5	11	9.1 (4.4)	7.8 (4)	4.6 (2.9)
	Absolute difference +30	11	39.2 (40.4)	39.7 (42.9)	31.2 (46.7)
	Fold change +30	11	3.5 (2.2)	3.2 (2)	2 (1)
T3	Pre	11	13 (6.7)	26 (11.5)	77.1 (58)
	Post +5	11	186.3 (122.9)^****^	198 (128.7)^****^	222.3 (116.9)^****^
	Post +30	11	64.7 (53.1)^****^	68 (52.5)^*^	96.7 (67.7)
	Absolute difference +5	11	173.2 (118)	172.1 (129.3)	145.3 (95.8)
	Fold change +5	11	14.6 (6)	8.5 (5.8)	3.7 (2.4)
	Absolute difference +30	11	51.7 (48.6)	42 (53.4)	19.6 (74.4)
	Fold change +30	11	4.8 (2.6)	2.9 (2.4)	1.7 (1.5)

*Note*: Values are presented as the mean (SD) in nanograms per millilitre. Significant differences from pre‐exercise values are labelled with asterisks as follows: *P < 0.05, ***P < 0.001 and ****P < 0.0001.

Abbreviation: LH, lithium–heparin.

**FIGURE 2:**
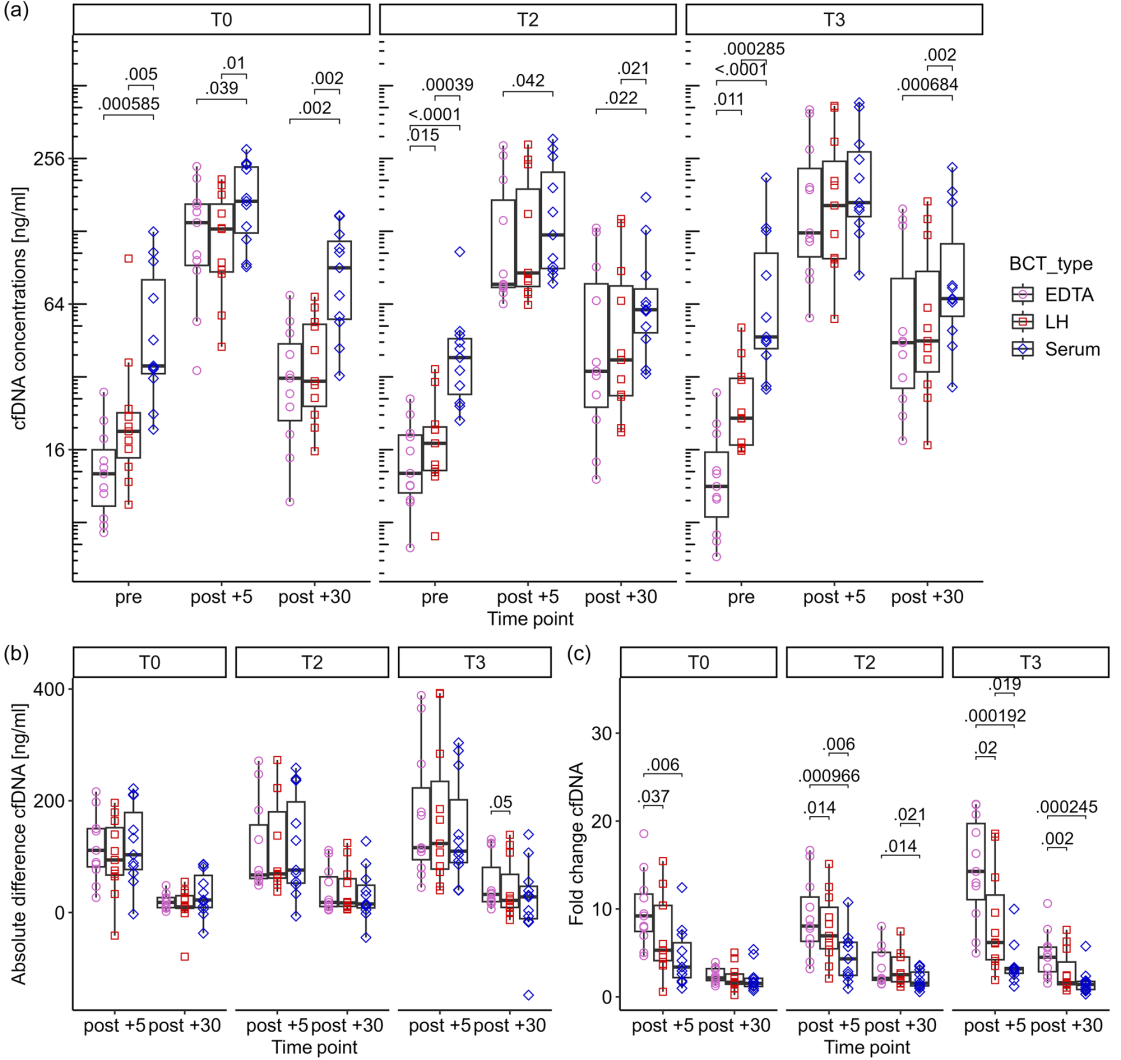
Impact of exercise on the cfDNA concentration in the different BCTs. (a) Absolute concentrations of cfDNA for the three exercise protocols. (b) Absolute differences in cfDNA compared with **pre‐exercise concentration**s for the three exercise protocols. (c) Fold changes of cfDNA compared with **pre‐exercise concentration**s for the three exercise protocols. Significant *P*‐values of ***post hoc*** comparisons with Bonferroni correction are labelled. Abbreviations: BCT, blood collection tube; cfDNA, cell‐free DNA.

A significant interaction effect of time, time point and BCT on cfDNA concentrations [*F*(2.86, 28.58) = 3.364, *P* = 0.034] was found. *Post hoc* comparison indicated that cfDNA concentrations in LH were significantly higher than concentrations in EDTA in the pre‐exercise concentrations of T2 and T3 (*P* = 0.015 and *P* = 0.011, respectively). Mean differences between LH and EDTA ranged between 3.4 and 13 ng/ml in the pre‐exercise values. Conversely, serum cfDNA concentrations were significantly higher than EDTA and LH at almost all time points (Figure 2a). Serum provided higher cfDNA concentrations than EDTA, ranging between 19.1 and 64 ng/ml at all time points. The cfDNA concentrations in serum were ∼15.2–51.8 ng/ml higher than in LH.

Next, we investigated whether there was an effect of the time, time point and BCT on absolute differences and fold changes (Figure 2b,c). For absolute differences, no differences were found when conducting repeated‐measures ANOVA [*F*(2.19, 21.87) = 0.791, *P* = 0.476]. With regard to fold changes, a significant effect of time, time point and BCT [*F*(4, 40) = 3.703, *P* = 0.012] was found. *Post hoc* comparison showed significant differences in 12 of 18 comparisons (Figure 2c). The cfDNA in EDTA provided higher fold changes than in LH, and the fold changes in EDTA and LH were higher than in serum, especially in the fold changes +5.

**FIGURE 3:**
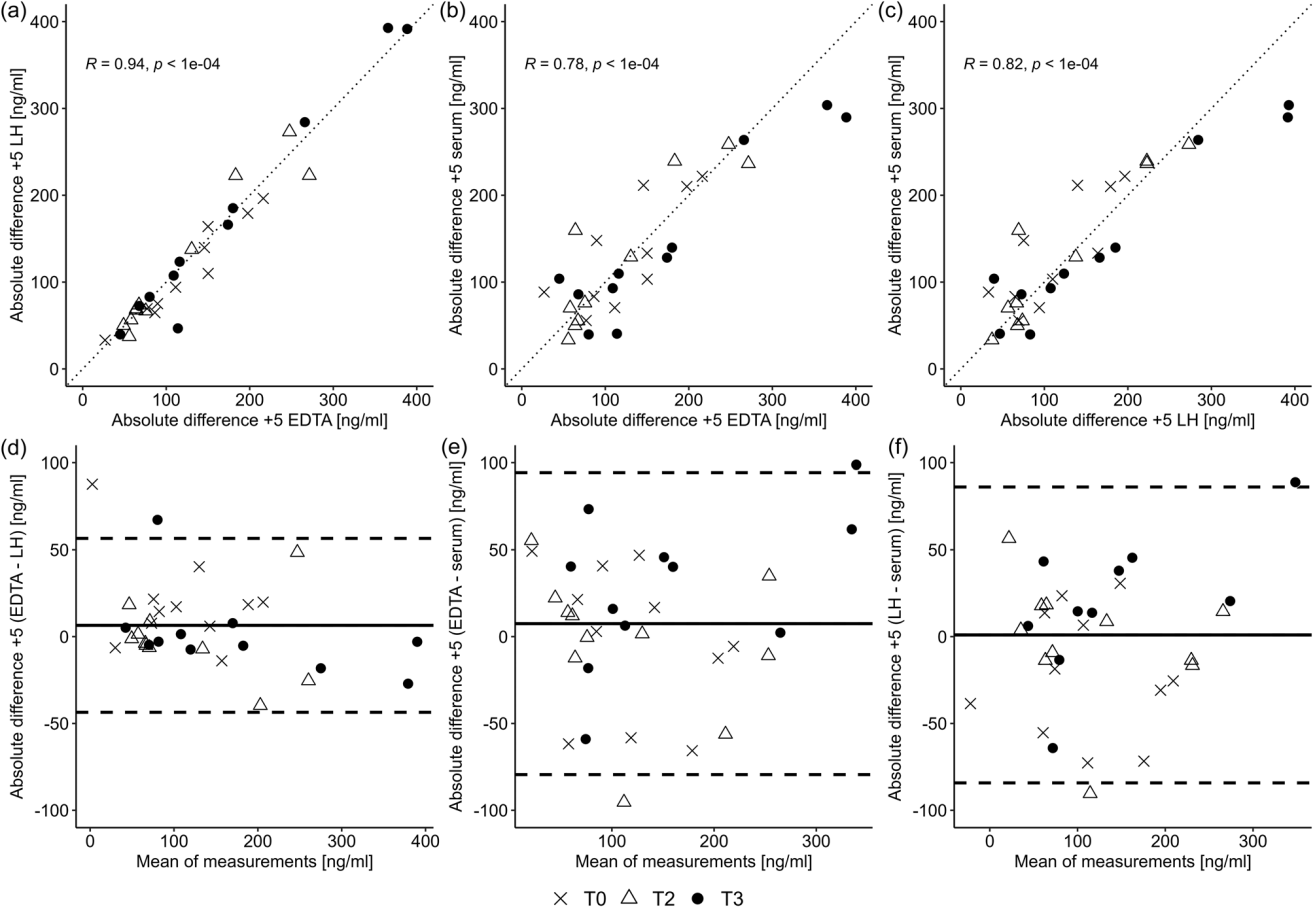
Scatter diagrams and Bland–Altman plots of cfDNA absolute differences +5. (a, d) Comparison between EDTA and LH. (b, e) Comparison between EDTA and serum. (c, f) Comparison between LH and serum. The dotted line in scatterplots is the line of equality. Continuous and dashed lines in Bland–Altman plots are the mean and the mean ± 1.96 SD. Abbreviations: cfDNA, cell‐free DNA; LH, lithium–heparin.

Absolute differences +5 were highly correlated for all BCTs (see Figure 3a–c). The strongest correlation was observed between EDTA and LH (*r*_s_ = 0.94, *P* < 0.0001), indicating that subjects with the largest absolute difference +5 in EDTA also exhibited the largest difference in LH. Likewise, absolute differences +5 in serum were strongly correlated with those in EDTA and LH (*r*_s_ = 0.78, *P* < 0.0001 and *r*_s_ = 0.82, *P* < 0.0001, respectively).

To examine the agreement between different BCTs regarding absolute differences +5, Bland–Altman analyses (Bland & Altman, 1986) were conducted (Figure 3d–f). Absolute differences +5 did not vary in any systematic way over the range of measurements. The comparison between EDTA and LH showed a mean bias of 6.5 (95% confidence interval: −2.6 to 15.6) ng/ml and the narrowest limits of agreement, ranging from −43.6 to 56.6 ng/ml. In contrast, comparisons involving serum showed wider limits of agreement. Absolute differences +5 in EDTA and serum demonstrated a mean bias of 7.5 (95% confidence interval: −8.2 to 23.2) ng/ml and limits of agreement between −79.4 and 94.4 ng/ml. Likewise, the comparison between LH and serum showed a mean bias of 1.0 (95% confidence interval: −14.4 to 16.3) ng/ml and limits of agreement ranging from −84.2 to 86.2 ng/ml.

### 3.3 Correlations of cfDNA with exercise‐related parameters

For a further evaluation of the cfDNA concentrations measured in the different BCTs, correlation analyses with exercise‐related parameters were calculated. At T0, positive correlations between absolute differences +5 in cfDNA and fold changes +5 in cfDNA with ${\dot{V}}_{O_{2}\mathrm{peak}}$, mean ${\dot{V}}_{O_{2}}$, energy expenditure and duration were observed. In detail, absolute differences +5 in cfDNA and the fold changes +5 showed strong positive correlations with the ${\dot{V}}_{O_{2}\mathrm{peak}}$, with cfDNA measured in EDTA (absolute difference: *r*_s_ = 0.72, *P* = 0.013; fold change: *r*_s_ = 0.90, *P* = 0.00016) and LH (absolute difference: *r*_s_ = 0.79, *P* = 0.0037; fold change: *r*_s_ = 0.86, *P* = 0.00061) expressing stronger correlations than serum (absolute difference: *r*_s_ = 0.75, *P* = 0.0073; fold change: *r*_s_ = 0.62, *P* = 0.043). Mean ${\dot{V}}_{O_{2}}$ values were correlated only with fold changes +5 in EDTA (*r_s_* = 0.81, *P* = 0.0026) and LH (*r*_s_ = 0.69, *P* = 0.019), but not with fold changes +5 in serum (*r*_s_ = 0.44, *P* = 0.18). The strongest correlations with energy expenditure were identified for fold changes +5 in EDTA (*r*_s_ = 0.91, *P* = 0.00011) and LH (*r*_s_ = 0.85, *P* = 0.001), second highest for absolute differences +5 in EDTA (*r_s_* = 0.70, *P* = 0.016), LH (*r*_s_ = 0.75, *P* = 0.0084) and serum (*r*_s_ = 0.65, *P* = 0.028), whereas fold changes +5 in serum were not correlated with energy expenditure (*r*_s_ = 0.55, *P* = 0.083). Additionally, only the fold changes +5 in EDTA were correlated with the duration of the all‐out test (*r*_s_ = 0.72, *P* = 0.013). In the endurance exercises at T2 and T3, positive correlations between cfDNA and ${\dot{V}}_{O_{2}\mathrm{peak}}$, energy expenditure and lactate were identified. Energy expenditure was correlated with absolute differences +5 and fold changes +5 in EDTA (absolute difference: *r*_s_ = 0.49, *P* = 0.019; fold change: *r_s_* = 0.49, *P* = 0.022) and LH (absolute difference: *r*_s_ = 0.44, *P* = 0.04; fold change: *r*_s_ = 0.44, *P* = 0.041). The ${\dot{V}}_{O_{2}\mathrm{peak}}$ was correlated with the absolute differences +5 in LH (*r*_s_ = 0.44, *P* = 0.038) and serum (*r*_s_ = 0.43, *P* = 0.047). Correlations with lactate were found in the fold changes +5 of LH (*r*_s_ = 0.52, *P* = 0.02) and serum (*r*_s_ = 0.49, *P* = 0.03). No correlations between cfDNA and mean RPE, mean body temperature or mean heart rate were observed.

## 4 DISCUSSION

Owing to the lack of standardization of cfDNA measurements in the exercise setting, the impact of pre‐analytical factors must be considered carefully to ensure comparable study results. Here, we evaluated the effects of different BCTs on cfDNA concentrations in standardized exercise protocols. Most importantly, we were able to demonstrate that cfDNA differences, rather than fold changes or absolute concentrations can be compared between BCTs, facilitating comparisons between exercise studies.

**FIGURE 4:**
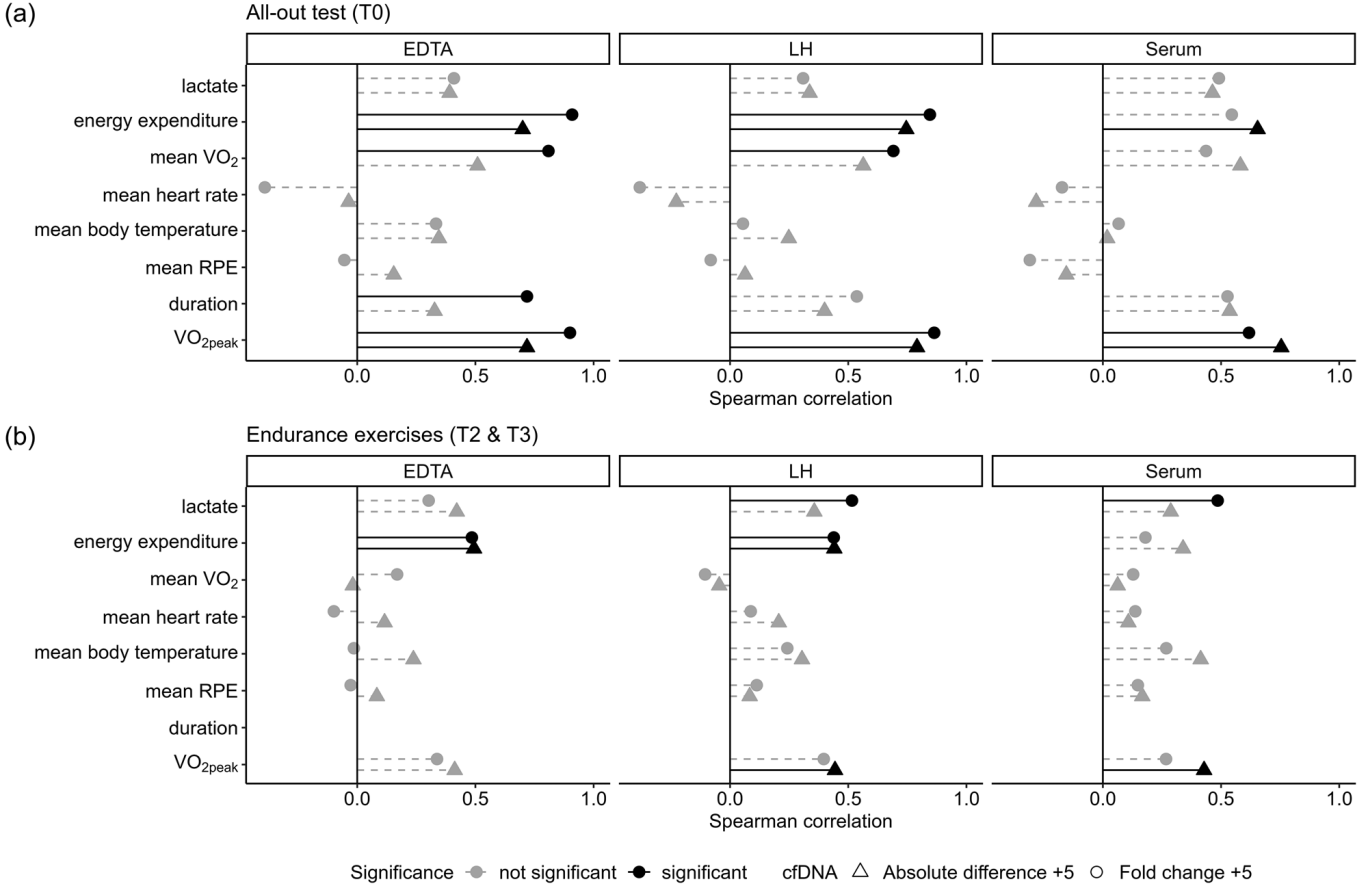
Spearman correlation between cfDNA and exercise parameters. (a) All‐out test (T0). (b) Endurance exercises (T2 and T3). Spearman correlation was conducted for the absolute difference +5 (triangles, absolute difference +5) and for the fold changes +5 (circles, fold change +5) in all BCTs. Significant correlations are depicted in black and non‐significant correlations in grey. The duration of endurance tests was constant for all subjects. Abbreviations: BCT, blood collection tube; cfDNA, cell‐free DNA; RPE, rating of perceived exertion; ${\dot{V}}_{O_{2}}$ **, oxygen uptake**; ${\dot{V}}_{O_{2}\mathrm{peak}}$ **, peak oxygen uptake**.

Different exercise modalities lead to marked increases in cfDNA. In a similar manner to other studies, we were able to demonstrate that cfDNA concentrations in all BCTs (LH, EDTA and serum) reflect the typical cfDNA kinetics observed during exercise protocols, which are characterized by low resting levels, marked increases during exercise and a return towards baseline levels 30 min post‐exercise. Notably, the BCT had a significant impact on the measured concentrations, whereby serum yielded higher concentrations than EDTA and LH plasma. The LH samples provided higher pre‐exercise concentrations than EDTA. Importantly, although the exercise‐induced cfDNA fold changes varied significantly between the BCTs, the absolute differences were consistent across all tubes. This highlights that differences in cfDNA are more comparable between studies using different BCTs. Exercise‐induced increases in cfDNA were correlated with energy expenditure, mean ${\dot{V}}_{O_{2}}$, lactate and ${\dot{V}}_{O_{2}\mathrm{peak}}$ (Figure 4). In contrast to previous studies in which LH inhibited the qPCR (Beutler et al., 1990; Jung et al., 1997; Yokota et al., 1999), LH plasma was shown to be suitable for cfDNA measurement in the present study. Even with direct quantification without cfDNA extraction, leaving LH residues in the final PCR mix, the PCR protocol was not inhibited. This demonstrates that, in appropriate analytical conditions, cfDNA can also be analysed in studies requiring LH for other analytes. The observed higher concentrations in serum compared with EDTA and LH align with previous studies (Ammerlaan & Betsou, 2019; Martignano, 2019; Trigg et al., 2018), which demonstrated higher cfDNA concentrations in serum in resting conditions. Importantly, we were able to demonstrate that the absolute differences from pre‐ to post‐exercise were comparable in all BCTs. This means that the observed higher concentrations in serum compared with EDTA and LH remained stable both before and after exercise and are independent of exercise‐induced changes. These findings suggest that the *ex vivo* DNA release during the clotting process in serum (Jung et al., 2003; Lee et al., 2001) occurs consistently, irrespective of exercise‐induced effects on blood homeostasis and coagulation (El‐Sayed et al., 2000; Posthuma et al., 2015). In both LH and EDTA plasma, the anticoagulants inhibit the clotting process, but they do so via distinct mechanisms, potentially influencing cfDNA concentrations in plasma differently. LH enhances the activity of antithrombin, leading to the inactivation of thrombin, a key enzyme in the coagulation cascade (Harmjanz, 2021). In contrast, EDTA acts by chelating divalent cations, such as copper ions and calcium ions (Guder, 2019). Given that calcium is a crucial cofactor in the coagulation process, its chelation inhibits the coagulation cascade (Arndt, 2019). Additionally, EDTA has a secondary effect that is important in the context of cfDNA measurements. By binding divalent cations, EDTA inhibits or even inactivates DNase in plasma (Ammerlaan & Betsou, 2019; Barra et al., 2015). DNase is the enzyme responsible for degrading cfDNA, and its inhibition by EDTA might enhance the stability of cfDNA in plasma. As our results indicate, this does not appear to affect the measured concentrations when the blood is processed immediately but might influence the outcome if there is a delay between venesection and processing.

With the Bland–Altman analyses, we were able to show that absolute differences +5 in EDTA and LH have the best agreement with one another. The absolute difference +5 for both BCTs is approximately the same. The majority of deviations are within a range of ∼50 ng/ml of the mean. Given the cfDNA differences from pre‐ to post‐exercise of up to 400 ng/ml in this study and the lack of clear cut‐off values for the comparability of cfDNA increases in the literature, this appears to be within an acceptable range. However, in contexts of lower exercise‐induced increases, these deviations must be viewed critically. For instance, in low‐intensity cycling (Mavropalias et al., 2021), where only marginal cfDNA increases are expected, the limits of agreement between different BCTs could exceed the pre‐ to post‐exercise differences. This issue is amplified further when comparing serum with either EDTA or LH, because deviations are significantly higher, particularly in cases where increases remain <100 ng/ml. Although the overall group showed comparable increases in cfDNA, comparing individual measurements using different BCTs appears problematic. Notably, the cfDNA measurement method itself can also show high deviations in some cases, even if the same sample is measured twice (Neuberger et al., 2021). It is therefore debatable whether the observed deviations in our study originate solely in the use of the different BCTs. To some extent, they could also be a consequence of assay imprecision itself. In our approach, we use direct quantification of cfDNA in plasma and serum. Isolating the cfDNA before quantifying it via qPCR might further increase the imprecision of the measurement (Devonshire et al., 2014).

Notably, the blood samples were obtained and processed in strict accordance with the National Cancer Institute guidelines (National Cancer Institute, 2020) and recommendations from reviews on pre‐analytical considerations of cfDNA measurements (Ammerlaan & Betsou, 2019; Bronkhorst et al., 2015; Ungerer et al., 2020). The time until centrifugation after blood sampling was reduced to a minimum of <5 min. The BCTs were carefully inverted immediately, to distribute the anticoagulants evenly. This could lead to lower external validity of our results when considering them in clinical settings, where longer transport distances and time until centrifugation are common, potentially resulting in damaging of blood cells and contamination with genomic DNA (Martignano, 2019). Still, there is evidence that cfDNA concentrations remain stable in all three BCTs with centrifugation within 2 h after venesection (Greytak et al., 2020). Some authors even proved stable values in EDTA up to 24 h (Trigg et al., 2018), whereas cfDNA concentrations in LH (Lam et al., 2004) and serum (Jung et al., 2003) seem to increase earlier. Further studies conducted in settings during clinical routine will provide more information on the comparability of BCTs for cfDNA analysis.

The informative value and the usability of a biomarker in the exercise context are strongly dependent upon its capacity to reflect the exercise load. Previous studies with EDTA samples could show that cfDNA is, amongst others, highly associated with energy expenditure, RPE, body temperature, duration, oxygen uptake, heart rate and lactate (Breitbach et al., 2014; Nogiec et al., 2024). The findings of this study indicate that cfDNA measured in EDTA and LH is, in general, more consistent with various exercise parameters than when measured in serum. In line with previous study results in EDTA plasma (Nogiec et al., 2024), we could demonstrate high correlations between exercise‐induced cfDNA and energy expenditure, especially in EDTA and LH. In contrast to findings by Breitbach et al. (2014), no correlations were observed between cfDNA and RPE, body temperature or heart rate in our study. This can be attributed to methodological differences, in that Breitbach et al. (2014) used multiple measurement points during the exercise test. The parallel increase in the repeatedly measured parameters might have caused a correlation. In our analysis, however, only a single post‐exercise time point was considered. By the inclusion of interval‐based exercise protocols in this study, we have demonstrated that correlations between cfDNA and exercise‐related parameters are not limited to all‐out or constant‐load protocols but also occur in different interval protocols. This expands the understanding of cfDNA dynamics, highlighting that the relationship between cfDNA and exercise‐related parameters is robust across various exercise modalities. Our findings emphasize the versatility of cfDNA as a biomarker in different exercise protocols and provide a basis for further exploration of its applications in diverse exercise settings.

The findings of this study, although focused on exercise physiology, might have broader implications for the use of cfDNA in clinical and diagnostic settings. The observed differences in the absolute concentrations between the BCTs, both at baseline and at elevated concentrations, are important considerations for other clinical applications, such as oncology (Song et al., 2022). Although the results based on cfDNA concentrations using qPCR might be transferable, differences could arise when other methodologies, such as sequencing or methylation analysis, are applied.

Although this study provides important insights into cfDNA quantification with different BCTs, some limitations must be acknowledged. Blood samples were always processed in a fixed order (serum, followed by LH, then EDTA) to minimize contamination, as recommended by the World Health Organization. Although this approach ensures that serum samples remain uncontaminated, it cannot fully exclude the possibility of a sequence effect influencing subsequent measurements in LH and EDTA. Although this order was necessary to avoid cross‐contamination, future studies might explore whether such effects impact the comparability of measurements across different BCTs. In our study, we did not address fragment length or integrity index of cfDNA, which is another limitation to consider. Previous research in EDTA plasma has indicated that physical exercise can influence cfDNA integrity (Stawski et al., 2019). The type of BCT used might affect not only cfDNA concentrations, but also the fragment lengths, such as through the release of genomic DNA fragments in serum. These are important considerations for future studies. This study included both male and female participants. Given that we did not control for the menstrual cycle phase or hormonal contraception in the female participants, the potential impact of the menstrual cycle warrants discussion (McNulty et al., 2020). This is particularly relevant because the exercise tests were conducted in different weeks, probably corresponding to different menstrual cycle phases. However, previous studies have shown that cfDNA concentrations do not vary significantly throughout the menstrual cycle, either at rest (Pölcher et al., 2010; Yuwono et al., 2021) or after exercise (Sawai et al., 2024). The sample size of our study was not sufficient to analyse potential differences between men and women reliably. Future research should therefore prioritize investigating sex‐specific influences on cfDNA dynamics, particularly focusing also on the influence of menstrual cycle phases. Based on the findings by Nogiec et al. (2024), who demonstrated that cfDNA released during different exercise modalities primarily originates from neutrophils, we assume that similar release mechanisms underlie acute increases in cfDNA across various settings. Therefore, it is plausible that the findings of this study are transferable to exercise modalities other than running. However, in cases of chronically elevated cfDNA levels observed in athletes, as described by Gentles et al. (2015), these mechanisms might differ. This distinction warrants further investigation to gain a better understanding of the dynamics of cfDNA release in chronic conditions.

## 5 CONCLUSION

The present study has demonstrated that direct quantification of cfDNA from various exercise settings can be conducted with EDTA, LH and serum BCTs. However, absolute concentrations differ across BCTs. To enable meaningful comparisons between studies using different BCTs, the focus should be on absolute differences rather than absolute concentrations or fold changes. Correlation analyses indicate stronger associations of cfDNA measured in EDTA and LH with exercise‐related parameters in comparison to serum. Therefore, EDTA or LH plasma appears to be more suitable for quantifying cfDNA concentrations in exercise studies. These findings might also have broader implications in clinical settings where cfDNA serves as a biomarker.

## AUTHOR CONTRIBUTIONS

Elmo W. I. Neuberger, Alexandra Brahmer, Barlo Hillen, Perikles Simon, Vincent Weber and Kira Enders were responsible for the conception or design of the work and for conducting the experiments. Kira Enders, Elmo W. I. Neuberger, Vincent Weber and Nils Haller were responsible for analysing and interpreting the data and drafting the work. All authors critically revised the work for important intellectual content. All authors have read and approved the final version of the manuscript and agree to be accountable for all aspects of the work in ensuring that questions related to the accuracy or integrity of any part of the work are appropriately investigated and resolved. All persons designated as authors qualify for authorship, and all those who qualify for authorship are listed.

## CONFLICT OF INTEREST

None declared

## Data Availability

The data that support the findings of the present study are available from the corresponding author on reasonable request.
